# Supplementary material for: Fatty Acid Amide Hydrolase Deficiency Is Associated with Deleterious Cardiac Effects after Myocardial Ischemia and Reperfusion in Mice
Source: Int J Mol Sci. 2022 Oct 21;23(20):12690. doi: 10.3390/ijms232012690 (PMC9604059; doi:10.3390/ijms232012690)
Supplement: Supplementary file 1 [file ijms-23-12690-s001.zip › legend to supp. figure s1.pdf]

Figure S1. The gating strategy for flow cytometry. (a) This analysis was performed as illustrated for myocardium. We defined the immune cells using CD45 staining versus forward scatter area. (b) The $CD45^+$ immune cells were further classified by Hoechst staining to differentiate living and (c) dead cells. (d) Next, we differentiated $CD4^+$ and (e) $CD8^+$ T cells and discriminated apoptosis according to Annexin V fluorescence. APC-A, allophycocyanin area; FITC-A, Fluorescein isothiocyanate area; FSC-A, forward scatter area.
