# Supplementary figures and images for: Fatty Acid Amide Hydrolase Deficiency Is Associated with Deleterious Cardiac Effects after Myocardial Ischemia and Reperfusion in Mice
Source: Int J Mol Sci. 2022 Oct 21;23(20):12690. doi: 10.3390/ijms232012690 (PMC9604059; doi:10.3390/ijms232012690)

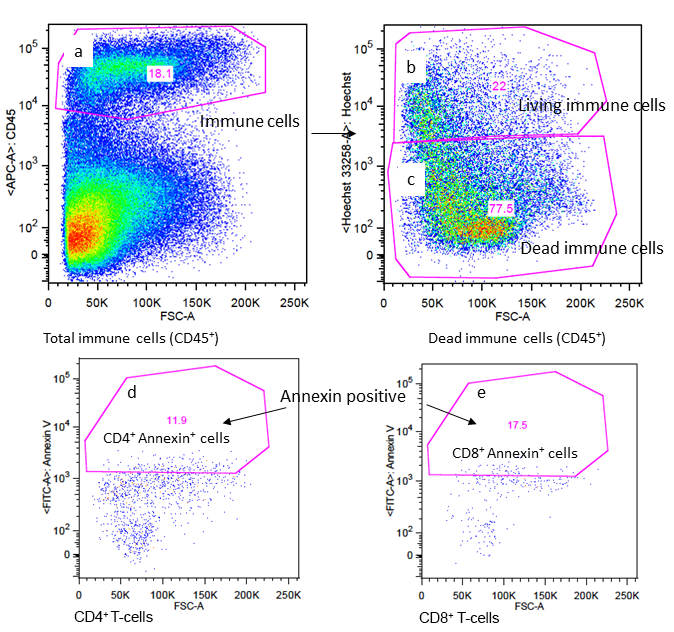

Supplement: Supplementary file 1 [file ijms-23-12690-s001.zip › Figure S1.tif]
